# An app-based physical activity intervention for people with hip and knee osteoarthritis: protocol for the PIANISSIMO feasibility study

**DOI:** 10.1186/s40814-025-01744-z

**Published:** 2025-12-05

**Authors:** Mathilde Mura, Berta Portugal, Caroline Mouton, Bernd Grimm, Romain Seil, Laurent Malisoux

**Affiliations:** 1 Physical Activity, Sport and Health (PASH), Department of Precision Health (DoPH), Luxembourg Institute of Health (LIH), Strassen, Luxembourg; 2 Department of Orthopaedic Surgery, Centre Hospitalier Luxembourg (CHL) — Clinique d’Eich, Luxembourg, Luxembourg; 3 Sports Medicine and Science (LIROMS), Luxembourg Institute of Research in Orthopaedics, Luxembourg, Luxembourg; 4 Human Motion, Orthopaedics, Sport Medicine and Digital Methods (HOSD), Department of Precision Health (DoPH), Luxembourg Institute of Health (LIH), Strassen, Luxembourg

**Keywords:** Osteoarthritis, Hip, Knee, mHealth, Physical activity, Health promotion, Healthy lifestyle

## Abstract

**Background:**

Osteoarthritis is one of the most prevalent musculoskeletal disorders. In osteoarthritis patients, physical activity has been shown to be an effective tool to improve quality of life as well as to reduce the pain associated with the disease and the development of additional comorbidities. Yet osteoarthritis patients often do not meet the level of physical activity recommended to stay in good health. The PIANISSIMO study primarily aims to test the adherence of people with hip and knee osteoarthritis to a 6-month intervention for the promotion of physical activity specific to this population using a dedicated mobile app.

**Methods:**

The PIANISSIMO study is a longitudinal, interventional feasibility study conducted through a dedicated mobile app. A total of 151 participants with hip or knee osteoarthritis will be included. The app will collect data and deliver the intervention. Data will be collected through questionnaires (i.e. demographic data, osteoarthritis history, sport history, pain and functional capacities, app evaluation) and from Apple Health/Google Fit apps. The PIANISSIMO study will use a 6-month physical activity intervention based on the *Capability, Opportunity, Motivation–Behaviour* change theory. Participants will receive a text notification on a daily basis; they will be asked to set their daily steps goal for the next week, and the mobile app will deliver feedback on daily step count and whether the goal of the week has been reached. The primary outcome of this study is the adherence (i.e. connection log and rate of answered questionnaires) to the app-based physical activity intervention. Retention will be calculated as the number of drop-outs over 6 months of follow-up. Moreover, the acceptability of the app and intervention by the study participants will be evaluated through a questionnaire.

**Discussion:**

This mobile app was designed to provide a digital solution for the promotion of physical activity in people with osteoarthritis. If the feasibility of delivering a physical activity intervention through the mobile app is confirmed, the efficiency of the tool in improving the quality of life of people with hip and knee osteoarthritis should then be properly investigated.

**Trial registration:**

Registration number

NCT06385028 (https://clinicaltrials.gov/study/NCT06385028)

Protocol version 1.2

**Supplementary Information:**

The online version contains supplementary material available at 10.1186/s40814-025-01744-z.

## Introduction

Osteoarthritis (OA) is the second most prevalent musculoskeletal disorder [1]. In 2019, it was reported that this degenerative condition mostly affected the hip and knee, with approximately 400 million prevalent cases in the world [2]. In 2017, Western Europe had an age-standardised prevalence rate of hip and knee osteoarthritis of 38.6 per 1000 inhabitants and an incidence rate of 2.01 per 100,000 person-years—reflecting increases of 7.2% and 8.0%, respectively, since 1990 [3]. A recent meta-analysis of 63 studies, involving 5397 knees, showed a prevalence of MRI-detected OA features ranging from 19 to 43% in adults over the age of 40 [4]. A study led in Germany found that the prevalence of people over 60 years old presenting with hip or knee OA was almost 22% [5]. This trend can be explained by the population ageing as well as an increased prevalence of obesity and traumatic injuries at an early stage of life, which will even further increase the OA burden in the next years [6–8].

The economic burden associated with OA is high for both patients and society, particularly due to work loss, premature retirement, disability and high health care costs [9]. Moreover, a recent meta-analysis has shown that hip and knee OA increase the risk of developing atherosclerosis and cardiovascular diseases [10]. Another meta-analysis showed that OA patients have higher risks of myocardial infarction or stroke compared with control subjects [11], likely due to a lack of physical activity [12].

In addition to being cardioprotective in the general population [13], the practice of physical activity has been shown to reduce symptoms of OA such as fatigue [14], depression [15] and pain [16], as well as increase quality of life [17]. In line with these benefits, the European Alliance of Associations for Rheumatology (EULAR) recommends physical activity, weight management and occasional painkillers as the first-line treatments for hip and knee OA [18]. The general physical activity recommendations, including cardiorespiratory fitness, muscle strength, flexibility and neuromotor performance, are considered to be feasible and safe for OA patients [3].

Pain is the most disabling symptom of OA, being the major driver of healthcare seeking and clinical decision-making [19]. Expensive and reactive treatments (i.e. analgesic drugs, joint replacement surgeries) are often favoured to overcome OA symptoms, and effective lifestyle behaviour strategies such as physical activity or weight loss are currently underused [20, 21]. Patients with OA actually often still believe that physical activity will further damage their joints; thus, they doubt the effectiveness of physical activity and have fatalistic beliefs about OA-related pain [22]. As a result, most patients with OA do not meet the levels of physical activity recommended to expect optimal health outcomes [23]. As low physical activity is associated with low physical capacities [24, 25], patients with OA often fall into this vicious circle where the lack of physical activity and capacities leads to the development of comorbidities [12].

The most recent EULAR guidelines for the care of OA recommend including behaviour change techniques in order to help patients go beyond the fear of pain and increase the long-term adhesion to an active lifestyle [18]. These behaviour change techniques have been identified by Michie et al. [26] as tools to implement the *Capability, Opportunity, Motivation–Behaviour* (COM-B) framework [27]. The COM-B framework states that behaviour change requires individuals to feel empowered with *capability* (i.e. physical and psychological abilities to perform the behaviour) and have the *opportunities* (i.e. social and psychological factors that enable the behaviour) in order to increase *motivation* (i.e. internal process that triggers the behaviour) [27]. Based on this framework and evidence from mobile health (mHealth) interventions, we identified that the most important behaviour change techniques for our target population are goal setting [28], educational content [29] and feedback on performance [30]. The challenge of a non-pharmacological therapy to promote a change of behaviour to a healthier and more active lifestyle is to overcome physical, psychological and social barriers. To achieve this goal, several recommendations have been made. First, knowledge about the practice of physical activity in patients with OA should be widely disseminated [20, 31]. Then, the effectiveness of these educational programmes’ dissemination should be improved by suggesting a wide range of options and opportunities to practice physical activity, in line with the WHO health campaign “Every move counts towards better health” [32]. Lastly, patients should be able to monitor their physical behaviour [20, 31]. Thus, self-monitoring and individual goal setting enable participants to tailor their daily steps goal to their current levels of fatigue and pain.

For most chronic conditions, physical activity interventions have been delivered by rehabilitation centres. However, for most patients, frequent trips are time-consuming and tedious [33, 34]. These logistical barriers could explain the low adherence to physical activity post-intervention [35], reducing the long-term benefits on health. In healthy participants, mHealth interventions have shown to be effective in increasing physical activity [36] and reducing sedentary behaviour [37]. A recent meta-analysis showed that feedback and monitoring, as well as goal planning, are efficient ways to promote a change in behaviour using mHealth interventions [38]. A preliminary study suggests that a 9-week web-delivered physical activity intervention is plausible, feasible and acceptable for patients with hip and knee OA [39]. The “bring your own device” assessments allow the use of participants’ own devices to record real-life data [40, 41]. The use of these digital interventions would allow increasing post-intervention adherence by implementing the behaviour (here physical activity) in the everyday life of the study participants during the intervention. Thus, this feasibility study will be conducted before implementing a large-scale trial aimed at increasing the physical activity behaviour of people with hip and/or knee osteoarthritis using an app. It will assess participant engagement toward the intervention by evaluating adherence, retention and acceptability to a 6-month intervention.

### Objectives

The PIANISSIMO feasibility study primarily aims to test the adherence of participants with hip and knee OA to a 6-month intervention for the promotion of physical activity using a dedicated mobile app.

The secondary aims are to assess the retention and acceptability of participants with hip and knee OA to the intervention.

Moreover, the study aims to investigate the daily steps pattern in participants with hip and knee OA during the 6-month intervention and to explore the relationship between physical activity and pain. Finally, physical activity patterns of people with hip and knee OA over the 6-month period following the end of the intervention will be analysed.

## Methods

### Study design

The PIANISSIMO study is a longitudinal, one-arm interventional, feasibility study funded by the *Oeuvre Nationale de Secours Grande-Duchesse Charlotte*. This protocol has been designed according to previously established guidelines [42]. The protocol has been written according to the adjusted version of the SPIRIT 2013 statement: Defining standard protocol items for clinical trials [43], including indication of Thabane and Lancaster [44], clarifying items of the CONSORT 2010 statement [45] regarding feasibility studies.

The entire study will be conducted through a dedicated mobile app specifically designed for the purpose of the study. The mobile app will collect data and deliver the intervention. Data will be collected through questionnaires (i.e. demographic data, OA history, sport history, pain and functional capacities, app evaluation) and from Apple Health/Google Fit apps. Adverse event (AE) can be reported at any time in the app. The intervention will not interfere with participants’ medical treatments, which will remain the decision of the participant in agreement with his physician. There is no financial compensation for trial participation.

### Study population

All participants must meet the following criteria to be eligible: Male or female aged ≥ 18 years old; diagnosed with any stage of hip or knee OA; live or work in Luxembourg; download and engage with the mobile app by fulfilling questionnaires; understand one of the following languages: English, French or German; and have a smartphone with internet connection. Presenting rheumatoid arthritis is the only exclusion criteria.

### Sample size

Assuming a minimum adherence rate of 67% in the last month, a 95% confidence level and an acceptable margin of error of 7.5%, a minimum of 151 participants with hip or knee OA is required.

### Recruitment

Advertisement for the PIANISSIMO study will be done in social media and by display of flyers in hospital and general practitioner offices. In both cases, a short explanation of the study will be followed by a QR code that will redirect participants to the App Store and Google Play Store to download the app. Then, the participants will access the detailed study protocol, participant’s rights for human clinical research and a data protection notice. The participants willing and able to agree to the terms of the study enrolment, as well as fulfilling eligibility criteria, will be included after the written informed consent form is electronically signed. A copy of these documents will be available for participants to consult in the mobile app during the study.

### Intervention

The PIANISSIMO study will use a 6-month physical activity intervention, based on the COM-B behaviour change theory [27], and including behaviour change techniques (BCT) extensively described in the literature [26]. The BCT used in the present study is detailed in Table 1.

**Table 1:** Behaviour change techniques used and mobile app-based intervention delivery

Behaviour change technique	Intervention
1. Provide information/instructions on the following:	Daily text notification
Consequences of behaviour *in general*	“Physical activity plays a role in hypertension control” “Physical activity helps to prevent falls and fall-related injuries”
Consequences of behaviour *to the individual*	“Physical activity WILL NOT damage your joints” “Physical activity can reduce depression in osteoarthritis patients”
Where and when to perform the behaviour	“Stand up and walk during TV commercials—go for a tea, a glass of water”; “Stretch for 5 min when you wake-up”
How to perform the behaviour	“Did you know gardening is physical activity?” “Stay hydrated! The effort will be less strenuous”
Time management	“If you plan it, you will do it. Schedule even short walks!” “At home or at work, set an alarm every 30 min to remind yourself to stand up. No need to go far!”
2. Fear arousal	Daily text notification “Inactivity is the 4th cause of premature death (before 70 years) and global mortality” “Low physical activity is associated with a worsening of pain and physical function”
3. Goal setting	Weekly goal setting in order to increase physical activity until the end of the 6-month intervention
4. Feedback on performance	Graphical representation of daily steps matched against the goal setting

The behaviour change techniques used in PIANISSIMO are based on [26]

First, participants will receive a text notification on a daily basis (except on Sunday). During the first week, notifications will mainly focus on how to practice physical activity, i.e. pointing out the participants’ capabilities and the opportunities to practice. The content of the notifications will then progressively address further their motivation, including knowledge about the health benefits of physical activity (a complete list of notifications is available in Additional file 1 ).

Second, participants will be asked to set their daily steps goal as their PA target for the next week every Sunday by a text notification. In case no response is given, another reminder will be set for the next day at 8 am. It is expected that participants will set a gradual daily steps goal, empowered by the knowledge they received and their increased PA capacities. Daily steps will be retrieved by an Application Programming Interface (API) in the Apple Health/Google Fit applications. The API centralises the data from the participants’ phone or from a connected watch, if a wearable device is connected to the phone. Thus, the PIANISSMO app will allow participants to follow their daily steps on a graph matched against their week’s goal.

Third, the mobile app will deliver feedback on daily step count and whether the goal of the week has been reached.

### Data collection

**Fig. 1:**
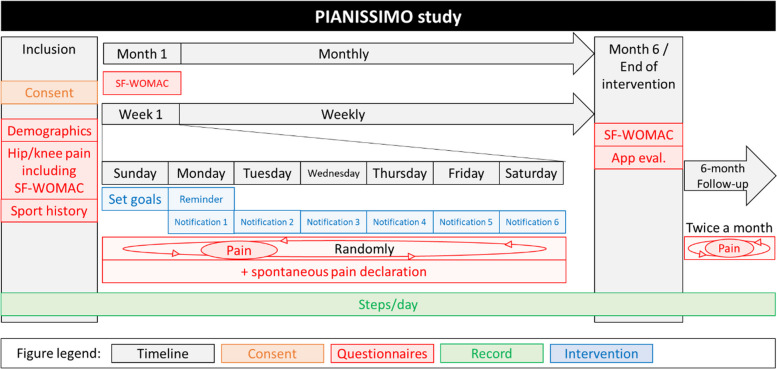
Study scheme of the PIANISSIMO study. SF-WOMAC, Short Form Western Ontario McMaster University Osteoarthritis Index

Figure 1 provides a detailed graphical representation of the data collection in the PIANISSIMO study.

### Data at inclusion

Participants’ demographics will be obtained at inclusion. This will include information on sex, age, height, weight, tobacco exposure (smoker, ex-smoker, never smoked), alcohol intake (doses per week), employment status (employed, unemployed, retired, not working, student), job category (list of minor groups of the International Standard Classification of Occupations [ISCO] classification), level of education (no formal education, primary education, lower secondary education, upper secondary education, post-secondary non-tertiary education, short cycle tertiary education, bachelor’s or equivalent, master’s or equivalent, doctoral or equivalent), annual income, country of residence, type of residence (apartment, house) and comorbidities (heart disease; hypertension; pulmonary disease; diabetes; ulcers or stomach disease; kidney disease; anaemia or blood disease; cancer; depression; OA, degenerative arthritis; back pain; rheumatoid arthritis—list from the self-administered comorbidity questionnaire [46]). OA will be characterised according to the affected joints (left/right knee, left/right hip, left/right foot, left/right elbow, left/right shoulder, left/right hand, spine), pain management treatment (analgesic levels 1, 2 or 3; physiotherapy), duration since diagnosis, mean of diagnosis (consultation only, consultation and imaging, imaging only), previous treatments (surgery, injections, thermal cure, physical activity) and symptomatic severity (assessed with a short form of the Western Ontario McMaster University Osteoarthritis Index [SF-WOMAC] [47, 48] described in detail below). Information on sports practice history will be collected including sport disciplines, frequency and duration of practice of previous and ongoing practice (Fig. 1).

### Follow-up data

Throughout the study, participants will be asked to complete a brief pain evaluation questionnaire that will be sent randomly once a week via the mobile app. In these questionnaires, participants will first be asked whether they have experienced any joint pain since the last time they completed the questionnaire. If they report pain, they will be asked to provide the following details: location of the pain (left/right knee, left/right hip, left/right foot, left/right elbow, left/right shoulder, left/right hand, spine), the intensity of the pain (rated on a Likert scale ranging from 0 [no pain at all] to 10 [worst pain ever]), any additional treatment used (analgesic levels 1, 2 or 3, cold application, alternative medicine), timing of the pain (wake up, morning, afternoon, evening, night), context of the pain (resting, walking exclusively, using the joint, carrying heavy loads) and the weather condition (rain, snow, cloudy, sunny) and perceived conditions (hot, mild, cold). They will also have the possibility to report a pain crisis at any time using the same questionnaire.

Participants will also be asked to answer the SF-WOMAC questionnaire [47, 48] monthly. This version comprises two parts, addressing pain and function, respectively. The first part comprises three specific questions addressing the average severity of the pain in the last 48 h while walking, stair climbing and resting. The second part addresses the average level of difficulty performing the functions during the last 48 h of descending stairs, ascending stairs, rising up from sitting, walking on flat, getting in/out of a car, going shopping, putting on socks and getting on/off the toilets. The participant will be asked to answer all 11 questions using a Likert scale ranging from 0 (none), 1 (slight), 2 (moderate), 3 (severe), to 4 (extreme).

At the end of the intervention, participants will be invited to give their feedback about the mobile app through a qualitative standardised questionnaire. Participants will be asked if the mobile app helped them increase their physical activity level, if they think they could maintain the actual level of physical activity without the intervention, what motivated them the most (goal setting, text notifications, both, nothing), if they liked the app and why, if they found that the app was adapted to their health condition, if they would recommend the app and how they heard about it. A free comment section will also be available. After 2 weeks without any interaction (i.e. no response to the questionnaire or connexion to the mobile app), the participant will be considered a drop out.

During the subsequent 6-month follow-up period, the pain evaluation questionnaire will be sent randomly twice a month (Fig. 1).

### Outcomes

#### Primary outcome

The primary outcome of this study is adherence to the mobile app-based physical activity intervention, measured using three indicators: the number of logins per week, the percentage of weekly goals set and the percentage of questionnaires completed by the participant during the 6 months. Adherence will be considered good if the number of logins per week does not decrease by more than 33% between the first and the last month and if at least 50% of the weekly goals are set and questionnaires are completed over the intervention.

### Secondary outcomes

Retention will be calculated as the number of dropouts over time during the 6 months. A retention of ≥ 33% of participants at the end of the 6 months will be considered a successful outcome. Moreover, the acceptability of the mobile app and the intervention by the study participants will be assessed qualitatively through a combination of open- and closed-ended questions. This will cover usefulness, most motivating features, user-friendliness, adaptability to condition, intent to continue physical activity and suggestions for improvement. Acceptability will be considered good if ≥ 70% of responses regarding usefulness, user-friendliness and adaptability to condition are positive. Feasibility will be validated if adherence, retention and acceptability meet the positive outcomes specified above.

### Exploratory outcomes

The physical activity pattern assessed by the evolution of daily steps throughout the 6-month intervention will be analysed. The bidirectional relationship between physical activity (e.g. cumulated weekly steps or peak in daily steps) and pain will be explored. Maintenance of the behaviour changes will be investigated during the 6 months following the intervention. All outcomes and their measurement period are presented in Table 2 and Fig. 1.

**Table 2:** Outcomes, their measurement and evaluation period

Outcomes	Measurement	Evaluation period				
		**Baseline**	**Weekly**	**Monthly**	**End of PA intervention (6th month)**	**Between the 6th and 12th months**
Adherence	Connection log	Continuously				
	Rate of goal set		x			
	Rate of questionnaire answered		x			x
Retention	No. of drop outs		x			x
Acceptability	Questionnaire				x	

*PA* physical activity

### Data management

Throughout the study, the sponsor of the project, the Luxembourg Institute of Health (contact: project manager), will be responsible for the monitoring of participants.

### Collecting and data management

It will be granted the protection of privacy and confidentiality to all participants by allocating a unique study identifier with participants’ inclusion number for data collection. The investigator will be responsible for maintaining the anonymity of each participant in the study. Information will be collected for each participant at a defined timeline (Table 2 and Fig. 1) and reported in a standardised clinical data structure (OpenEHR) automatically filled out with data collected by the app. The source documents include the original data extracted from the app that will be stored in the standardised clinical data structure by LIH investigators. The investigator will be the sole person allowed to authorise access to the study source data during monitoring, audits or inspection visits. Data from the PIANISSIMO app will be extracted and stored on a LIH password-protected computer that can be accessed only by registered investigators (M. M., L. M., G. B.). All raw data will be checked monthly by the investigator to ensure that all protocols and ethical guidelines for data collection and analysis are followed. The data manager will use computerised consistency tests in order to flag the presence of non-standard, missing, aberrant or inconsistent data throughout the data transfer process. Study data will be entered through the standardised clinical data structure hosted on OpenEHR software, which allows for real-time data quality control. An interim analysis will be done on the data stored on OpenEHR after half of the participants finish the study (*n* = 75) and again after every additional 20 participants. In order to meet the regulatory requirements, the OpenEHR software complies with the recommendations concerning computerised systems for the management of clinical trials and electronic signatures and standards. Connection to such a server is made through the use of a unique password and identifier specific to each user, which will only give access to participant data. The maintenance and security of the database will be the responsibility of the study’s sponsor. An audit function will be integrated into the software, allowing traceability of the date of data collection as well as any further modifications completed. The encrypted data will then be transmitted to the department responsible for data management via a secure Internet connexion.

### Monitoring and quality control

An independent data and safety monitoring board will be formed for any potential issues and will be operated by the study sponsor. Members will not have any financial or scientific conflicts of interest with the PIANISSIMO trial. The monitoring board will be composed of researchers and/or clinicians with experience in clinical trial monitoring. The objective of the monitoring board will be to ensure the safety of trial participants as well as maintain the scientific integrity of the trial by monitoring the data. The monitoring board will meet if any serious adverse event related to the intervention happens. After every monitoring visit, a monitoring report will be prepared by the monitoring committee. If this report warrants a protocol change, an amendment will be drafted for the ethic committee to approve. The clinical trial registry will be amended accordingly. There are no predefined statistical guidelines for the premature termination of the study. As the risk to develop serious adverse events linked to the intervention is low, no steering committee or audit is planned.

### Statistical analysis

Data management and analysis will be performed using R software (version 2023.6.1.524). Descriptive statistics will be computed to describe baseline participants’ sociodemographic, feasibility and acceptability data. Frequencies and percentages will be reported for categorical variables and means and standard deviations for continuous variables. The evolution of daily step count over time, as well as the bidirectional relationship between daily step count and pain, will be explored using linear mixed-effects models. Open-ended question responses will be analysed using a qualitative content analysis inductive approach as described by Elo [49].

## Discussion

This feasibility study aims to test the adherence, retention rates and acceptability of delivering an intervention for the promotion of physical activity using a dedicated mobile app among people with hip and knee OA.

Currently, more than 100 applications are suggested as answers to the search “physical activity” or “walk” on the App store, but the vast majority of them focus on exercise rather than physical activity. Furthermore, the search returns no results when “osteoarthritis” is added to these keywords. As this population needs specific advice and motivation compared to the general population, a dedicated app is needed for physical activity promotion in people with hip and knee OA.

This study is innovative in several aspects. Firstly, it will be carried out in Luxembourg, a country where eHealth is only just beginning to develop, despite the massive use of smartphones by its inhabitants (i.e. more than 94% of them own a smartphone [50]). Moreover, the aim of this mobile app is not to make financial profit. Thus, its design and creation were not bound to the emergency to be quickly released and profitable but were meticulously designed to ensure that the app is functional and scientifically accurate. Data and analysis generated by this study will be used to plan further fully powered trials such as randomised controlled trials. In addition, for health care professionals, this type of mobile app can be an aid to provide physical activity to people with OA in their daily lives. Evidence from the cardiac rehabilitation field shows that home-based interventions can increase adherence to physical activity in comparison with rehabilitation centre interventions [51]. In addition, offering remote physical activity interventions can reduce the healthcare burden, offering a solution to the shortage of health care professionals or to their lack of knowledge in physical activity. For the people with OA, an increase in daily steps can result in positive outcomes on physical, psychological and social health.

This study also has some limitations. Research-grade physical activity monitors using accelerometers are considered the gold standard to measure physical activity in daily life. However, such devices are expensive; they cannot deliver live feedback, and cannot be used for periods longer than 2 weeks. Thus, they are mainly used in research. Here, we choose a pragmatic approach in order to simulate more real-world settings in mobile app usage. The daily step count measured by the smartphone or connected watch was chosen to assess adherence to the physical activity intervention. Although it is not considered the gold standard, and its validity can be questionable [52], a large part of the population owns a smartphone with or without an associated connected watch. Thus, this real-life based approach was favoured.

The use of the mobile app will hopefully promote more active behaviour in people with hip or knee OA. The results of this study will be released in peer-reviewed journals and presented in sport science and orthopaedics international conferences. These results will represent a first step into digital health and will be useful to plan more robust studies.

## Supplementary Information

Additional file 1. Complete list of notifications send to the participants.

Additional file 2.

## Data Availability

Not applicable.

## Abbreviations

COM-B: Capability, Opportunity, Motivation–Behaviour
EULAR: European alliance of associations for rheumatology
mHealth: Mobile health
OA: Osteoarthritis
PIANISSIMO: Personalised physIcal Activity promotioN in osteoarthrItis patientS uSIng a sMartphone-based sOlution
SF-WOMAC: Short Form Western Ontario and McMaster Universities Arthritis Index
WHO: World Health Organization
