# Supplementary material for: An app-based physical activity intervention for people with hip and knee osteoarthritis: protocol for the PIANISSIMO feasibility study
Source: Pilot Feasibility Stud. 2025 Dec 5;12:5. doi: 10.1186/s40814-025-01744-z (PMC12797945; doi:10.1186/s40814-025-01744-z)
Supplement: Supplementary file 1 — Additional file 1. Complete list of notifications send to the participants. [file 40814_2025_1744_MOESM1_ESM.pdf]

Additional file 1: Complete list of notifications send to the participants.

Weeks	N°	Notification
1	1-1	Physical activity is not necessarily sport! Any activity with movement counts!
	1-2	Did you know that walking is physical activity? Get around on foot!
	1-3	You can do physical activity whenever you want! At work, at home, on your travels…
	1-4	Go to the bakery or the post office on foot
	1-5	To be healthy, the human body needs to move!
	1-6	What if you catch up with a friend while walking this weekend?
2	2-1	Exercise does NOT only mean go to the gym! You could be more active around your home or workplace
	2-2	Did you know that climbing the stairs is physical activity?
	2-3	Start by systematically going down the stairs instead of taking the elevator
	2-4	Pain may not be related to your physical activity at all, it does NOT mean permanent damage
	2-5	Set manageable and measurable goals (and track them on Apple health, Google fit)
	2-6	Did you know housework is physical activity? What if you dusted your home?
3	3-1	Get out of the bus/tram one stop early and walk the rest of the way
	3-2	Take the stairs instead of the lift at least once a day
	3-3	You can go around the block before you pick up your mail
	3-4	Sedentary behaviour is spending time sitting or lying down
	3-5	WHO recommends 150min of moderate physical activity or 75min of intense physical activity /week + muscle strengthening
	3-6	Let's not be too sedentary this weekend, go out and enjoy some fresh air!
4	4-1	At work, go to the rest room on another floor
	4-2	It is important to break up sedentary behaviour, a few steps are enough to break the routine!
	4-3	Walk the dog, or a neighbour's dog. You will both be happier afterwards!
	4-4	Physical activity WILL NOT damage your joints further
	4-5	Only 5% of adults perform sufficient physical activity to protect their health
	4-6	Grab your coat and enjoy a short walk even if the weather is bad
5	5-1	Stand up and walk after every book chapter - go for a tea, a glass of water
	5-2	You can have an unhealthy sedentary behaviour at home, at work, be wary of your screen time!
	5-3	Do a small shop on foot (Post, pharmacy)
	5-4	Inactivity is when you do not meet the physical activity recommendations
	5-5	If you plan it, you will do it. Schedule even short walks!
	5-6	You can walk or cycle to do your shopping
6	6-1	Get out of the lift one or two floors down and climb the stair!
	6-2	Minimise your sitting time and stand up as often as possible
	6-3	Light or mild pain does not worsen osteoarthritis
	6-4	Exercising can improve your mood, make you more focused and make you feel stronger mentally.
	6-5	Inactivity + sedentary behaviour = accumulation of cardiovascular risk
	6-6	What if you went dancing this weekend to boost your physical activity?
7	7-1	Watching TV on the couch is considered sedentary behaviour
	7-2	Take a longer route to go the bakery/grocery store
	7-3	Join a local association for group sessions

	7-4	Sport is an activity with performance objective (e.g. basketball). There are other ways of being active!
	7-5	Sedentary behaviour is harmful for health even if you are active
	7-6	You have an errand to run this weekend? Check on a map if you can go on foot!
8	8-1	At home or at work, set an alarm every 30min to remind yourself to stand up. No need to go far!
	8-2	Stay hydrated! The effort will be less strenuous
	8-3	Physical activity can help you manage the pain linked to osteoarthritis
	8-4	Aerobic exercise is a moderated exercise you can maintain for an extend period of time (walking, gardening)
	8-5	Inform your friends and family you want to be more active and get support from them
	8-6	You can plan a family walk this weekend
9	9-1	Bad weather is not an issue, you can climb stairs even on a rainy day!
	9-2	Stretch for 5min when you wake-up
	9-3	Sitting in motorised transportation is considered to be sedentary behaviour
	9-4	Choose an activity you enjoy or that you are good at (gardening, shopping, biking, visit cities…)
	9-5	The results are not always immediate, so be patient, the benefits will soon be felt.
	9-6	Choose active activities with (grand-)children instead of sedentary ones
10	10-1	Walk at a pace you are comfortable with! No need to go too fast
	10-2	Favour walking, cycling and public transport for travel and commuting
	10-3	Discomfort during or after physical activity DOES NOT necessarily mean injury (especially at the beginning)
	10-4	Cardio respiratory fitness is the body's capacity to transport and use oxygen, it is associated with good health
	10-5	The higher your cardio respiratory fitness, the easier the effort will be !
	10-6	Water- and land-based activities are just as effective in osteoarthritis patients. How about a trip to the pool?
11	11-1	If you live close enough, go to work on foot once a week
	11-2	Listen to an audiobook to keep you entertained while walking
	11-3	It is normal to feel sore sometimes, especially if you haven't exercised in a while !
	11-4	Did not reach your goals yesterday? No worries, today is a brand new day!
	11-5	Invite your friends and family to adopt more active behaviour with you
	11-6	Some video games can allow you to be active with your (grand-)children
12	12-1	Incorporate physical activity into your daily routine, for example walk to work, or climb the stairs
	12-2	Go for a walk around the block after dinner
	12-3	Physical activity may reduce fatigue linked to osteoarthritis
	12-4	Low physical activity is associated with a worsening of pain and physical function
	12-5	Physical activity improves quality of life in osteoarthritis patients
	12-6	Visit a friend on foot or by bike
13	13-1	Walk 5 more minutes than usual
	13-2	Electric bikes make it easier to get around and count as physical activity!
	13-3	Physical activity improves strength in osteoarthritis patients and the general population
	13-4	Physical activity can reduce depression in osteoarthritis patients
	13-5	WHO recommends at least 7500 steps/d for patients with chronic diseases such as osteoarthritis

	13-6	Break the routine and do your usual walk in the other direction!
14	14-1	Park your car further away from the shop entrance or at work
	14-2	Physical activity can ease articular stiffness
	14-3	During moderate physical activity you can hold a basic conversation (difficulty 5-6/10)
	14-4	Physical activity prevents the risk of falls in osteoarthritis patients
	14-5	Movement is the main treatment of arthritis according to the most recent guidelines
	14-6	What if you went shopping to meet your steps/day goal?
15	15-1	Sitting at your desk is considered to be sedentary behaviour
	15-2	Walking can help avoid unreliable public transport
	15-3	Physical abilities (strength, stamina, …) define your physical condition
	15-4	Physical activity helps prevent falls and fall-related injuries
	15-5	Everyone has their own pace! Don't compare yourself to others and stay focused on your goals
	15-6	Go to the museum, you will walk without realising it
16	16-1	You can cycle on an ergometer in front of the TV
	16-2	Physical abilities (strength, stamina, …) define your physical condition
	16-3	Being physically active can increase your fitness and your chances of living longer
	16-4	Moderated physical activity reduces the risk of cardiovascular related mortality, even if you don't practice sports
	16-5	Physical inactivity is linked to obesity
	16-6	Visit a city or a castle, you will walk without realising it
17	17-1	Walk during casual or professional phone calls
	17-2	You can do two lighter trips instead of one, to empty the car
	17-3	Physical activity improves cardiorespiratory fitness in osteoarthritis patients
	17-4	Getting up off the couch can reduce your cardiovascular risk
	17-5	Decreased aerobic capacity is associated with increased risk of cardiovascular death, but is reversible with activity at any age
	17-6	Book a restaurant close enough to get there on foot
18	18-1	You can walk slowly when you are on an escalator
	18-2	The more you move, the less difficult it becomes
	18-3	Walking can help you lose weight
	18-4	Moderated physical activity reduces the risk of cancer related mortality, even if you don't practice sports
	18-5	Physical activity can reduce blood pressure and the risk of hypertension
	18-6	Organise a picnic and go for a walk this weekend
19	19-1	Walk with comfortable shoes. You'll be happier!
	19-2	During intense physical activity it is almost impossible to talk (difficulty > 7/10)
	19-3	You can take breaks or slow down, no need to walk continuously
	19-4	Physical activity can reduce the risk of ischaemic heart disease
	19-5	Recreational running does not cause osteoarthritis!
	19-6	You can plan an electric bike trip this weekend!
20	20-1	Listen to music to keep you entertained while walking
	20-2	You could walk or bike: in urban areas, 1km = 4 min by car or bike and 15min on foot
	20-3	Being physically active helps you to age better, even if you were not active previously!
	20-4	Practice of physical activity appears to be even more beneficial for health in high income countries (than low income countries)
	20-5	WHO recommends physical activity for healthy ageing

	20-6	Did you know gardening is physical activity? You could do some this weekend!
21	21-1	Order your taxi one block further and walk to the meeting point
	21-2	Warm up and cool down gradually to minimise pain during exercise
	21-3	Being physically active, you are about one third less likely to be restricted in your daily activities or walking in older age
	21-4	Listens to your body - reduce but don't stop activity during a flair up, start again as soon as you are comfortable
	21-5	Inactivity is the 4th cause of premature death (before 70y/o) and global mortality
	21-6	This weekend, reach your objectives with your friends or family!
22	22-1	Stand up and walk during TV commercials - go for a tea, a glass of water
	22-2	Over time, your body strengthens and soreness will decrease
	22-3	Physical activity is important for joint support and fitness
	22-4	Moderated physical activity reduces the risk of total mortality, even if you don't practice sports
	22-5	Enjoying the activity is the most important part
	22-6	Organise a visit to a place you love!
23	23-1	Physical activity improves joint range of motion and mobility
	23-2	The benefits of physical activity increase with age
	23-3	Moderate physical activity is beneficial for sleep quality
	23-4	Physical activity can prevent premature death
	23-5	However out of shape you think you are, there's still time to improve your fitness.
	23-6	Organise a visit to a place you have never been to!
24	24-1	Improving your physical capacity could make everyday tasks easier
	24-2	Exercise produces endorphins. Endorphins make you happy!
	24-3	Physical activity can reduce the risk of ischaemic stroke
	24-4	Physical activity is associated with better quality of life
	24-5	Be a role model to inspire your family/friends to move!
	24-6	No need to go far, Luxembourg has some nice parks and landscapes to visit
25	25-1	Being physically active reduces the number of sick days related to osteoarthritis
	25-2	Sedentary behaviour is associated with increased pain and loss of physical function
	25-3	Physical activity can reduce the risk of developing diabetes
	25-4	Physical activity promotes self-esteem, social support and self-efficacy
	25-5	Physical activity can reduce the incidence of cognitive decline
	25-6	Before going on a walk check for difficulty. Many walks are easier than you might think!
26	26-1	Physical activity reinforces the muscles that support your joints
	26-2	Physical activity does not exacerbate pain or fatigue linked to osteoarthritis
	26-3	Physical activity plays a role in hypertension control
	26-4	Walks can help you to reduce your stress
	26-5	No adverse events have been reported in physical activity osteoarthritis studies
	26-6	Well done, you have completed the program !
